# The Promising Role of Selenium and Yeast in the Fight Against Protein Amyloidosis

**DOI:** 10.1007/s12011-024-04245-x

**Published:** 2024-06-03

**Authors:** Marek Kieliszek, Katsiaryna Sapazhenkava

**Affiliations:** Department of Food Biotechnology and Microbiology, Institute of Food Sciences, Warsaw University of Life Sciences—SGGW, Nowoursynowska 159 C, Warsaw, 02-776 Poland

**Keywords:** Selenium, Yeast, Selenite sodium, Amyloids, Protein aggregation, Food protein

## Abstract

In recent years, increasing attention has been paid to research on diseases related to the deposition of misfolded proteins (amyloids) in various organs. Moreover, modern scientists emphasise the importance of selenium as a bioelement necessary for the proper functioning of living organisms. The inorganic form of selenium—sodium selenite (redox-active)—can prevent the formation of an insoluble polymer in proteins. It is very important to undertake tasks aimed at understanding the mechanisms of action of this element in inhibiting the formation of various types of amyloid. Furthermore, yeast cells play an important role in this matter as a eukaryotic model organism, which is intensively used in molecular research on protein amyloidosis. Due to the lack of appropriate treatment in the general population, the problem of amyloidosis remains unsolved. This extracellular accumulation of amyloid is one of the main factors responsible for the occurrence of Alzheimer’s disease. The review presented here contains scientific information discussing a brief description of the possibility of amyloid formation in cells and the use of selenium as a factor preventing the formation of these protein aggregates. Recent studies have shown that the yeast model can be successfully used as a eukaryotic organism in biotechnological research aimed at understanding the essence of the entire amyloidosis process. Understanding the mechanisms that regulate the reaction of yeast to selenium and the phenomenon of amyloidosis is important in the aetiology and pathogenesis of various disease states. Therefore, it is imperative to conduct further research and analysis aimed at explaining and confirming the role of selenium in the processes of protein misfolding disorders. The rest of the article discusses the characteristics of food protein amyloidosis and their use in the food industry. During such tests, their toxicity is checked because not all food proteins can produce amyloid that is toxic to cells. It should also be noted that a moderate diet is beneficial for the corresponding disease relief caused by amyloidosis.

## Introduction

**Fig. 1:**
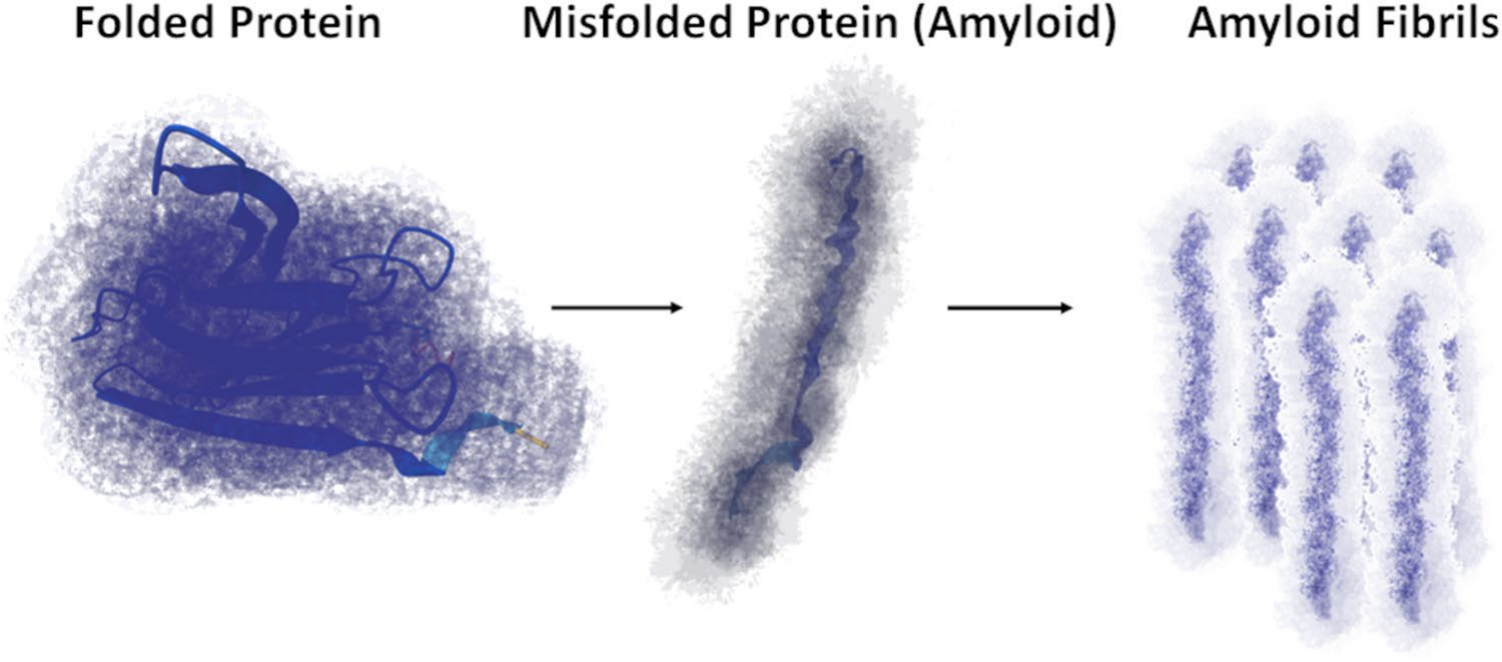
Scheme of amyloid formation

The development of diseases related to amyloidosis (Aβ) has been the subject of intensive research for many years. Currently, the mystery of this disease, which causes damage to organs and tissues and thus the appearance of various clinical symptoms, is not yet fully solved [1]. Amyloidosis involves the deposition of misfolded proteins (amyloids) in various organs and is associated with neurodegenerative diseases in humans [2], such as Alzheimer’s disease (AD), Parkinson’s, and Huntington’s, and may lead to the development of cancer. It should be noted that so far, the aetiology of these diseases is not well understood. After a certain period of time, proteins found in a healthy body break down and are replaced by new ones. However, amyloid protein forms permanent deposits [3]. It is worth emphasising that a properly constructed protein takes the form of twisted chains [4]. During the course of the disease, for unclear reasons, this structure destabilises and partially unfolds, as a result of which the proteins form a non-covalent cross-β polymer with fibres (Fig. 1). These polymers accumulate in organs where disease-specific damage occurs. An organ that contains large amyloid deposits loses its elasticity and biomechanical properties [5]. Insoluble amyloid deposits damage tissues and organs and prevent them from functioning properly [6, 7]. Misfolded proteins can form in various ways, leading to amyloidosis. Moreover, damaged and misfolded proteins may interact undesirably with other molecules and form aggregates in cells. Proteins may have an internal tendency to change conformation, which manifests itself with age. Another mechanism is the replacement of a single amino acid in the protein, which may result in the destabilisation of the structure [8]. The next mechanism is the proteolytic remodelling of the protein precursor, as in the case of the β-amyloid precursor protein (APP) in Alzheimer’s disease. According to Maślińska et al. [9], the aggregation of the main precursor protein in the form of fibrils is accompanied by the deposition in all deposits of a pentagonal glycoprotein from the serum called the P component (serum amyloid protein (SAP)). The mechanisms presented may occur independently or in connection with each other. In addition to the intrinsic amyloidogenic potential of the pathogenic protein, other factors may also synergistically participate in amyloid deposition. For example, the protein precursor must reach a critical concentration locally to induce fibril formation, and this process may be enhanced by environmental factors and interactions with specific substances found in the extracellular matrix [10]. Amyloid monomers and oligomers may have a direct toxic effect on cell viability [11, 12]. Amyloidogenic monomers can become incorporated into the cell membrane, damaging its permeability or creating pores that disturb the function of mitochondria and cytoplasmic reticulum [13]. Additionally, amyloid fibrils in the extracellular matrix may enhance the aggregation [14] and abnormal conformation of neighbouring precursor proteins, causing the effect of self-replication of subsequent amyloid structures [15]. An example is the formation of a fibrinogen coat on cancer cells, which can be prevented by administering a redox-active form of selenium [16]. Selenite can interfere with the formation of parafibrin (fibrin-like polymer fibres) by oxidising sulphydryls to disulphides in fibrinogen [17]. This element is considered one of the most important elements, playing a key role in human health. Selenium deficiency leads to many pathological processes and diseases, including cancer [18]. It has been shown that the selenium atom is contained in selenocysteine, which is necessary for the functioning of several enzymes [19]. Moreover, enriching the diet with selenium leads to the inhibition of oncogenesis and reduces the risk of cancer [20]. This may be due to the fact that various forms of this element have antioxidant properties, which help prevent many diseases [21, 22]. Research on selenium in various fields of medicine shows complex and sometimes contradictory results regarding its impact on the inhibition of cancer [23, 24]. According to Rayman [25], a non-linear relationship exists between selenium status and cancer. Similarly, as reported by Hatfield et al. [26], several studies did not establish any anticancer or chemoprotective effects of selenium. However, the research presented by Kim et al. [27] showed that selenium inhibits the development of cancer by blocking the expression of matrix metalloproteinases (MMP). Additionally, selenium can increase the level of interleukins (e.g. IL-2, IL-4, IL-22), which has a beneficial effect on the regulation of immune functions [28]. According to Xia et al. [20], selenium can influence the body’s immune functions by inhibiting the nuclear factor NF-κB, regulating the Nrf2 transcription factor and ferroptosis. These functions of selenium are mainly related to the mechanisms of oxidation and reduction of metabolites of this element to simple compounds that have the greatest protective effect and strong antioxidant properties [27]. Selenium-containing proteins show high potential in preventing the development of Alzheimer’s disease. Antioxidant stress and reversible regulation of redox proteins are among the most important biological functions of selenoproteins [29, 30]. Selenoproteins, exemplified by TXNRD and MSRB1, have an essential role in regulating redox activity and restoring immune cells damaged by oxidative stress [28]. These properties endow selenium with a strong potential to prevent the accumulation of β-amyloid, a misfolded protein produced during inflammation associated with Alzheimer’s disease [31].

Yeast is also used to study the patterns of many disease states in the human body. It is very important to see yeast cells as an opportunity to understand the underlying biological mechanisms and develop methods to fight various diseases. Over the past few years, yeast has emerged as an excellent model for molecular studies related to various neurodegenerative disorders caused by the misfolding of pathogenic proteins [32]. The ability to determine and study gene expression in yeast cells became more important when it was discovered that human genes have orthologs in the yeast genome. Such results make it possible to conduct research on yeast homologues of human genes, which is of key importance in understanding the mechanism of occurrence of particular disease states, including amyloidosis and cancer.

In this article, we discuss the influence of selenium and yeast cells on the process by which protein aggregates are created, along with a short description of how food proteins may be involved in the development of amyloidosis. The presented scientific topic is of great interest to many research centres whose goal is to understand the functioning of processes important in the formation of protein aggregates. However, it should be stated that the continuous development of this topic is a key element that will contribute to expanding knowledge in this area. Consequently, such activities will allow us to propose new directions for further scientific research aimed at explaining this scientific problem and its impact on the metabolic mechanisms occurring in cells.

## Protein Amyloidosis

**Fig. 2:**
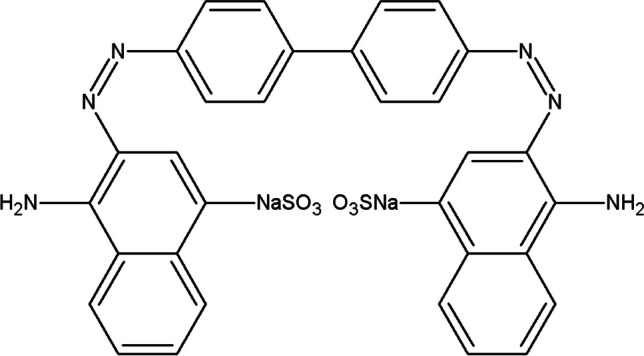
Structural formula of the dye—Congo red

Amyloidoses are a heterogeneous group of diseases characterised by extracellular accumulation of insoluble fibrous proteins called amyloids in tissues and organs [33]. Amyloid is a protein polymer found in tissue deposits as unbranched, stiff fibrils [34]. These fibres are detected by Congo red staining (Fig. 2), which results in a green or yellow colour [35]. About 20 proteins form amyloid deposits in vivo in humans, which may be associated with Alzheimer’s disease [16]. Amyloids may arise as a result of persistent chronic inflammation or when there is a high concentration of the precursor protein in the blood serum [36, 37]. Another cause may be mutations in the genetic material.

### Amyloidosis Formation

The formation of amyloid may be influenced by the presence of the amyloid protein variant in the serum, as well as by genetic and environmental factors [38]. There are two types of amyloidosis: systemic and localised. Systemic amyloidosis occurs when a fibrous protein precursor is released into the blood plasma upon expression in a soluble form, where it then accumulates as amyloid in the extracellular space [39]. A characteristic feature of localised amyloidosis is the deposition of amyloid deposits in cells that synthesise precursor proteins [40]. It should be emphasised that the model of amyloid formation that is common to all types of proteins is not yet fully understood. However, simplified methods for creating fibrous protein forms have been proposed [41, 42]. The formation of amyloid fibres involves the transformation of proteins into their structural form, which is only partially unfolded [43, 44]. Proteins with a variable conformational structure are more willing to combine and form amyloidosis fibres. Such protein structures interact with each other, increasing their tendency to aggregate and form protein oligomers. The mutual formation and combination of oligomers, as well as the final shape of protein aggregates in a characteristic molecular environment, are also influenced by the presence of specific physical and chemical factors [45, 46]. Some fibrils have elongated linear structures, usually 10–20 nm wide. Many of these fibrils contain protein subunits that are repeatedly arranged along the axis to form intersecting β-sheet structures [47]. It is worth noting that the degree of protein phosphorylation also plays a decisive role in the amyloidosis process [48]. The formation of such protein structures is a key stage in the development of amyloidosis, which is why it is so important to know the individual factors influencing their formation.

### Types of Amyloidosis

There are four types of amyloidosis, and over 36 different types of proteins have been discovered that cause the deposition of amyloid deposits [49]. The type of amyloidosis depends on the type of protein accumulated. Each amyloid type name is referred to as the letter A, followed by the abbreviation of the protein type [49]: amyloid light chain—primary (AL), serum amyloid A protein—secondary (AA), transthyretin amyloidosis (ATTR), and wild-type ATTR. AL amyloidosis is characterised by the deposition of monoclonal immunoglobulin light chain fibres that are released by the proliferation of an abnormal clone of plasma cells. During the development of AL amyloidosis, the secondary structure of the light chain of the abnormal monoclonal antibody changes, which in turn results in an unstable conformation. Misfolded antibodies then combine into monomers and form amyloid fibres [44]. AA amyloidosis is the most common type of amyloidosis in the world, with most cases associated with chronic inflammation of the body [49, 50]. It is characterised by the production of serum amyloid A protein, which is a high-density apolipoprotein and serves as a dynamic acute phase reagent. Amyloid A synthesis occurs in hepatocytes as a precursor in response to transcriptional stimuli from various pro-inflammatory cytokines, i.e. interleukin (IL)-6, IL-1 and tumour necrosis factor (TNF) alpha [51].

ATTR amyloidosis is associated with an inherited disease that is caused by the production of a mutant protein called transthyretin (TTR) [52]. TTR is a protein that consists of four monomers forming a tetramer and is produced by the liver. TTR participates in the transport of thyroxine and retinol. During TTR mutations, there is a weaker interaction of monomers, which leads to monomer dissociation. This, in turn, leads to the folding of monomers and then aggregation into amyloid fibres.

### Pathology of Amyloidosis

Amyloidosis was first described by Rokitansky in 1842 [53]. However, the term amyloidosis was first used by Rudolf Virchow (1852) to describe extracellular deposits of insoluble protein material showing a starch-like reaction with iodine and sulphuric acid [54]. The aetiology of amyloidosis remains unclear [55]. Additionally, there are theories linking the disease with disorders in the immune system [56]. Many different mutations have been identified, but the most common of them is the transthyretin mutation—Val122Ie, which is associated with cardiomyopathy [49]. Wild-type amyloidosis is associated with ATTR, and the transthyretin protein produced is not mutated. This type of amyloidosis is caused by the accumulation of amyloid in the heart. With age, the TTR tetramer may lead to the release of misfolded intermediates and subsequently form amyloid deposits [57]. The formation of misfolded proteins may be associated with neuroinflammation, which leads to many neurodegenerative diseases, including Alzheimer’s disease. The most important causal pathway leading to neuroinflammation is believed to be dysregulation of iron levels [58]. Dysregulation may be indicated by high serum ferritin levels, which in turn negatively affects the structure of red blood cells and may lead to abnormal blood clotting. The causes of iron level dysregulation may be related to oxidative stress, nutritional stress, pharmacological stress, and mechanical damage. This dysregulation may lead to the reactivation of dormant microorganisms that a person may have acquired during previous infections or dysbiosis [59, 60]. When microbiologically balanced, their growth is limited by the lack of free iron. These microorganisms are capable of producing highly inflammatory allergens, i.e. lipopolysaccharides (LPS) [61]. LPS found in the outer wall of gram-negative bacteria have a high ability to produce large amounts of amyloids. Allergen production and amyloid formation are strong inflammatory activators and cytokine inducers, which then lead to the formation of free radicals and affect vascular permeability [62]. Subsequently, this may lead to coagulopathy, for example, amyloidogenic blood clotting, which in turn has a greater chance of initiating the cell death process [61]. Table 1 shows examples of amyloid proteins and their precursors.

**Table 1:** Examples of amyloid proteins and their precursors

Amyloid protein	Precursor	Syndrome or involved tissues
ATTR	Transthyretin	Heart, blood vessels
ALys	Lysozyme	Kidneys, liver, spleen
B2M	Beta-2 microglobulin	Kidneys
ACys	Cystain C	Brain
APro	Prolactin	Pituitary
ALac	Lactoferrin	Cornea
AA	Serum amyloid	Organs in the central nervous system
AFib	Fibrinogen	Kidneys

Moreover, the production of these allergens indicates that human physiology may be chronically exposed to a wide range of amyloid loads, which in turn may lead to various age-related diseases as the gastrointestinal epithelium and the blood-brain barrier become significantly restructured and permeable [63]. The first symptoms causing the onset of age-related disease, for example, Alzheimer’s disease (AD), are damage to cerebral vessels, altered blood flow to the brain, and abnormal haemostasis [64]. Misfolded protein, β-amyloid, is the main cause of Alzheimer’s disease [65]. This amyloid accumulates in the blood vessels of the brain, which in turn leads to degenerative vascular changes that play a key role in AD [66].

The mechanism by which β-amyloid alters thrombosis and haemostasis is not fully understood [67, 68]. During blood clotting, it is necessary to convert fibrinogen into insoluble fibrin [69]. High levels of fibrinogen are thought to be associated with an increased risk of Alzheimer’s disease. β-amyloid induces the formation of abnormal, resistant blood clots, which in turn can lead to the accumulation of fibrinogen, causing inflammation [70].

## Characteristics of Selenium

Currently, one of the most important and intensively researched microelements is selenium. This element was discovered in 1817 by the Swedish chemist J.J. Berzelius [71] during research on a new method of producing sulphuric acid. During the combustion of sulphur, a red-brown precipitate obtained from pyrites was observed (iron sulphide) from a mine in Falun (Sweden). Initially, this characteristic sediment was considered the most toxic compound—arsenic, so the processing of Falun pyrite was avoided. However, the phenomenon was considered interesting and was analysed again. During subsequent tests, it was found that the sediment contained a new, previously unknown compound with properties similar to tellurium. Drawing on the similarity to tellurium, whose name in Greek means Earth (Tellus), selenium was so named after the Greek word for Moon [72].

The demand for selenium and its content in the body is small, but this amount is necessary for life and proper functioning. Moreover, selenium is an element for which there is little difference between the recommended amount and the toxic dose. According to Hadrup and Ravn-Haren [73], the normal level of intake of this element is from 11 to 280 µg Se/day (0.15 to 4 µg Se/kg body weight/day). Data provided by the EFSA Panel on Nutrition [74] states that the tolerable upper level of selenium intake for adults should be 255 µg/day. It is worth noting that intervention studies conducted in populations with low selenium content have shown a beneficial effect of this element on health [73]. However, there is still a lack of data on the relationship between its dose and cellular response [73, 75]. Therefore, further research on the health effects of dietary selenium should be conducted to establish appropriate reference values. Products rich in selenium include meat, brazil nuts, yeast, seafood, cereal products, garlic, and legumes [76]. Selenium has antioxidant and pro-oxidant properties, depending on its concentration [77]. In the inorganic form, it occurs in the form of selenate (VI) and selenite (IV) [78], while in the organic form, it consists of two basic amino acids—selenomethionine and selenocysteine [79]. Both amino acids are created by replacing the sulphur atom present in cysteine and methionine with a selenium atom [80]. All inorganic forms of selenium can be converted to its organic form in eukaryotes, which will then be incorporated into selenoproteins [81].

The very high biological activity of selenium in the human body is due to the presence of this element in many selenoproteins, which are necessary for maintaining redox homeostasis, and the course and regulation of many important biochemical processes [29]. So far, over 25 genes responsible for the biosynthesis of selenoproteins have been identified in mammals [82, 83], including 3 families of selenoenzymes important for life processes: glutathione peroxidases, thioredoxin reductases, and iodothyronine deiodinases. The active centres of these enzymes contain selenocysteine [84], which is considered to be the 21st protein amino acid encoded by the UGA codon [85]. The low pKa (5.2) may be responsible for the catalytic properties of selenocysteine [86]. The redox potential of selenocysteine is lower than that of cysteine, and as a consequence, selenocysteine is easily oxidised to form a selenocysteine dimer. The second amino acid, selenomethionine (SeMet), acts as a scavenger of reactive oxygen species in cells [87]. SeMet supplementation has been proven to protect against NH_3_-induced apoptosis by inhibiting oxidative stress in the endoplasmic reticulum in the kidneys [88].

Selenoproteins take part in protecting cells against the effects of reactive oxygen species, oxidative damage, and maintaining proper body immunity [89]. However, it should be emphasised that the health-promoting effect of selenium depends on the type of cells examined, the combinations of this element, and its concentration.

There are publications suggesting that selenium has exceptional and beneficial effects on antioxidant and immune processes [28, 90, 91]. In addition, selenium has an immunostimulating effect. The antibody response to infection can be enhanced by the administration of selenium, which can increase circulating levels of immunoglobulins and immune complexes, affecting lymphoid cell proliferation [92]. The interaction of selenium protects organs against the harmful effects of free radicals [93, 94]. Due to the antioxidant properties of selenium, the risk of cancer is reduced, and its anticancer properties have been recognised, although the mechanisms behind these effects are not fully understood [28]. The effect of selenium on cancer cells depends on its chemical form, dose, type of cancer, and degree of bioavailability. Taking supplementation in the form of selenium yeast reduces the incidence of cancer in various organs [21, 28, 95]. To sum up, it should be noted that selenium has a number of properties that are potentially important in the prevention of many diseases. However, current scientific reports are insufficient to clearly assess the role of this element in various therapies. This is due to the existence of many chemical forms of selenium, each of which is characterised by different biological properties. Therefore, further research and development work should be carried out to clarify the in-depth role of this element in eukaryotic cells.

### Selenium and Its Effect on the Inhibition of Amyloidosis

Due to the growing pharmacological and medical interest in selenium compounds with potential therapeutic properties, it is increasingly the subject of many scientific studies. There are also indications that selenium may be helpful in the prevention and treatment of diseases related to amyloidosis. An example is neurodegenerative diseases, which most often develop as a result of oxidative stress. Due to its antioxidant properties, this element is considered a potential therapeutic agent. Moreover, selenium plays an important role in the proper functioning of the brain [96]. The human body selectively retains selenium in brain tissue, even during periods of selenium deficiency [97, 98]. Low selenium levels throughout life may be associated with the development of neurodegenerative diseases, such as Alzheimer’s disease (AD). AD is associated with loss of memory, speech, and object recognition, subsequently leading to executive and behavioural impairments.

This element is also widely used in the prevention of metabolic diseases, such as type 2 diabetes [99, 100]. A characteristicz feature of diabetes is chronic hyperglycemia, leading to disturbances in the metabolism of proteins, carbohydrates, and fats [101]. During the development of type 2 diabetes in humans, amylin is produced, which is the main component of amyloid deposits in the pancreas [102, 103]. Amylin is a peptide hormone consisting of 37 amino acids [104]. This hormone is produced in the islets of Langerhans in the pancreas and secreted into the blood along with insulin. The formation of amyloid in the islets of Langerhans leads to impaired activity of pancreatic cells producing insulin, which in turn leads to an increase in blood glucose. The relationship between the formation of amyloid amylin and the development of type 2 diabetes is not fully known. In a study conducted by Mirhashemi and Shahabaddin [105], it was shown that selenium leads to the inhibition of the formation of amyloid deposits. The mechanism by which selenium inhibits the formation of amyloid amylin deposits may be related to the destabilisation of the intramolecular disulphide bridge present in the amyloidogenic amylin. These bridges may determine the formation of amyloid [105]. It is worth noting that selenium inhibits the aggregation of human islet amyloid polypeptide (hIAPP) by 11% [106]. Phycocyanin obtained from spirulina containing selenium (Se-PC) may affect the inhibition of the formation of reactive oxygen species (ROS) and hIAPP fibrils by interfering with the combination of peptides [107].

**Fig. 3:**
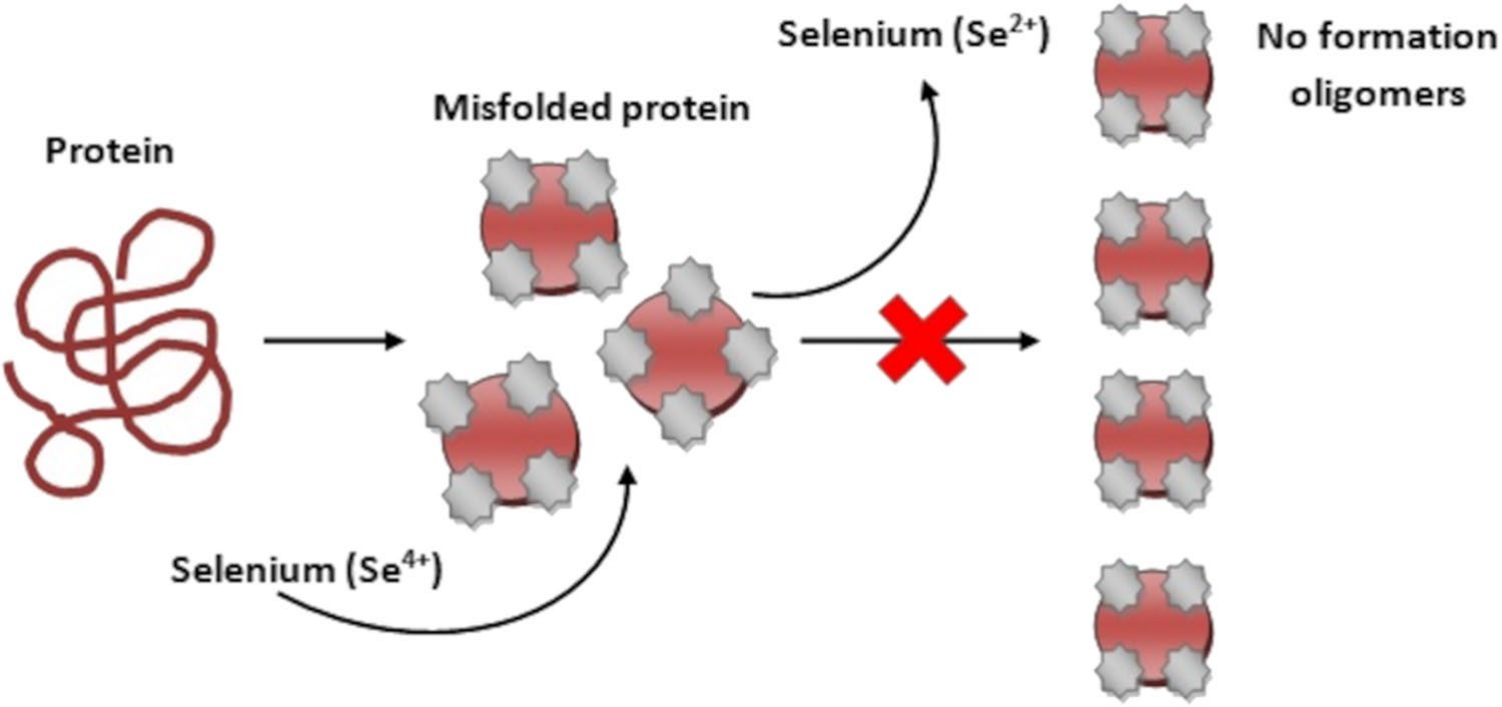
Inhibitory effect of selenium

Previous research conducted using selenium has shown its important role in preventing the formation of amyloids. Figure 3 shows the proposed action mechanism of selenium on amyloid fibres. Parafibrin fibres are completely resistant to degradation by proteolytic enzymes. The attachment of parafibrin to the cell surface causes inflammation [108]. Selenite (Na_2_SeO_3_) can interfere with the formation of polymer fibres called parafibrin. Its oxidising properties, especially in reactions with sulphydryl groups (thiol, –SH), disrupt the interactions of protein disulphide bridges. The consequence of the oxidation of thiol groups to disulphides is a disruption of the interaction of fibrinogen with plasma proteins, thus preventing the formation of a fibrous structure [21, 109]. Currently, the exact mechanism of the beneficial role of selenium in inhibiting the formation of amyloids is not clear. One possible explanation is that the decrease in selenium levels in the body corresponds to a decrease in selenoproteins, which protect against oxidative stress [110].

According to the information provided by Luo et al. [111], selenium has the property of inhibiting γ-secretase activity. In addition, the element reduces the expression of amyloid precursor protein and the production of Aβ. These processes result in selenium’s protective effect against β-amyloid neurotoxicity. Moreover, the presence of higher levels of selenium in serum reduces the occurrence of two isoforms of β-amyloid (i.e. Aβ42 and Aβ40). According to Varikasuvu et al. [112], selenium levels in Alzheimer’s patients are reduced. The consequence of this is reduced antioxidant capacity and increased oxidative stress in cells. Research presented by Zhang et al. [113] found that Se-methylselenocysteine (Se-MetSeCys) increased the expression and enzymatic activity of thioredoxin reductase. Furthermore, Se-MetSeCys reduced tau protein (MAPT) phosphorylation by inhibiting the activity of glycogen synthase kinase-3 beta (GSK-3β). The use of inorganic selenium compounds (sodium selenate) inhibited the hyperphosphorylation of tau protein by regulating the activity of protein phosphatase 2 (PP2A) [114]. Such processes inhibit the formation of twisted paired aggregates of these protein fibrils, the defective conformation of which may cause neurodegenerative effects. It is worth noting that selenium nanoparticles (SeNP) coated with peptides have the ability to bind and inhibit the formation of Aβ40 fibrils [115]. Saini et al. [116] showed that the use of selenium nanoparticles and silymarin (SLY-XG-Se) in neuronal cell lines alleviated cytotoxicity induced by Aβ1–42. Additionally, it was shown that such nanoparticles stabilised with exopolysaccharide xanthan gum can be used in the therapy of neurodegenerative diseases. The research results presented by Ramshini and Rostami [117] showed that the use of SeNP inhibits the aggregation of hen egg-white lysozyme (HEWL) amyloid. The obtained research results may be promising in the treatment of Alzheimer’s disease. Similar research results were shown by Vicente-Zurdo et al. [118]. The authors found that chitosan-stabilised selenium nanoparticles (Ch-SeNPs) reduced the aggregation of β-amyloid. Selenium nanoparticles (SeNPs) exhibit various physicochemical properties because they can have chemotherapeutic, anticancer, and antibacterial properties. In another study, Vincente-Zurdo et al. [119] described that selenium nanoparticles have the ability to reduce Aβ aggregation by changing the hydrophobic and electrostatic interactions occurring between fibrillar forms of proteins. This technology using nanoparticles may be helpful in determining the changes that occur during the induction of the formation of pathological fibrillar fibres. Such processes using selenium may be a key tool in the future to investigate the inhibition of the protein aggregation process. Research conducted by Cruz et al. [120] showed that the use of selenium nanoparticles can inhibit the formation of amyloidosis fibres. Moreover, they have very strong anti-lung cancer properties. In recent years, quantum dots have gained particular interest among researchers. Guo et al. [121] synthesised selenium quantum dots (Se QDs), which, thanks to their very small size and excellent biocompatibility, can quickly penetrate the blood-brain barrier. The authors found that Se QDs showed strong free radical scavenging activity and could protect cells against damage caused by oxidative stress. Se QDs can not only inhibit Aβ aggregation but also reduce Aβ-dependent cytotoxicity. In the future, selenium quantum dots could be used to develop new therapeutic strategies to prevent the development of neurodegenerative diseases. Nevertheless, the exact mechanism of action of Se QDs still requires detailed research. Zhou et al. [122] showed that the use of selenium quantum dots showed inhibition of β-amyloid aggregation. According to the authors, this could be due to the presence of α-carboxyl and amino groups on the edge of Se QDs, which influence the occurrence of multivalent interactions with Aβ. Vivash et al. [123] found that selenium supplementation by AD patients improved insulin homeostasis and slowed down the development of the disease. It can be concluded that selenium has a strong affinity for emerging amyloid fibres, changing their electrostatic and hydrophobic bonds. The consequence of these processes is a reduction in amyloid aggregation and a reduction in the occurrence of oxidative stress [124]. It is worth emphasising here that the molecular mechanisms underlying the neuroprotective effect of selenium on the formation of β-amyloid remain unknown. Therefore, the current understanding of the physiological role of selenium in individual stages of the pathogenesis of diseases caused by amyloidosis requires further scientific research.

To sum up, it should be stated that due to the mechanism responsible for the ability to inhibit amyloidosis or the functioning of selenoproteins, it should be assumed that selenium does not work only through one mechanism. There are probably several of them, and they work together. Future studies conducted by different research teams should demonstrate which of the mechanisms plays the most important role.

## Yeast in Research on Amyloidosis and Other Disease States

Various yeast species are used as model organisms in many fields of molecular biology and genetics. The term ‘yeast’ often suggests a reference to *Saccharomyces cerevisiae*. This species is currently the most frequently used model organism in various scientific studies [125]. The yeast *S. cerevisiae* is the first eukaryotic organism whose genome has been fully sequenced and then safely used as an effective tool and model for studying DNA repair, ageing, gene expression, and molecular and cellular pathways of human diseases, including cancer [126, 127]. The features that have made budding yeast a biological model used in hundreds of research centres around the world include its fast doubling time, ease of genetic manipulation, low breeding costs, small genome (about 200 times smaller than the human genome), and the ability to easily monitor the cell cycle progression through nuclear and cellular morphology. In addition, it is possible to conduct research on recessive mutations using *S. cerevisiae*, which occur in haploid and diploid states during their life cycle. Recessive mutations do not always have disease symptoms because, in the diploid state, they may be masked by the wild-type allele [128].

Currently, more and more discoveries are being made using yeast, an example of which is the production of a wide range of human proteins and heterologous proteins for structural and functional studies. This, in turn, leads to the discovery of the mechanisms of cancer and other various human diseases using the model organism *S. cerevisiae*. Moreover, research on *S. cerevisiae* helps to understand the process of development of various diseases, such as ataxia-telangiectasia syndrome, type 2 diabetes, and hereditary colorectal cancer [129, 130]. There are two groups of diseases whose causes are studied in yeast: diseases caused by human genes that have orthologs in *S. cerevisiae* and diseases in which there are no orthologs in the yeast genome [131].

In recent years, the incidence of neurodegenerative diseases (ND) has been increasing significantly among people around the world. ND are complex and characterised by neuronal loss coupled with the formation of misfolded proteins, leading to the formation of amyloid aggregates in the central nervous system. Therefore, the use of *S. cerevisiae* as an experimental model organism was essential in uncovering the mechanisms of many key disease processes associated with ND [132].

Yeast has the ability to bind various elements on the surface of the cell wall or accumulate them inside the cell in organic connections, mainly with proteins or enzymes [133, 134]. The effectiveness of the selenium absorption process, i.e. bioavailability, depends largely on the individual characteristics of each organism, but also on the chemical form in which this element was introduced. The availability of organic forms of selenium depends largely on the digestibility of proteins containing this element. According to Marson et al. [135], yeast is a source of easily digestible protein. Therefore, these microorganisms enriched with selenium are an effective, safe, and natural source of selenium and are the most absorbable form of this element [136, 137]. In accordance with European Union legislation (no. 427/2013) [138], selenite, sodium selenate, and selenium yeast are permitted as selenium supplements. Furthermore, the selenium content in selenium yeast can range from 0.2 to 0.35 mg/kg in accordance with Commission Implementing Regulation (EU) no. 2017/2233 [139]. Importantly, yeast, exemplified by *S. cerevisiae*, is currently an ideal model for studying human neurodegenerative diseases, because many signalling pathways and proteins associated with human neurological diseases are conserved in this microorganism [132, 140]. Taking into account the safety of using yeast, they can be effectively used to discover the functioning of complex mechanisms, e.g. the activity of β- and γ-secretase, which are the main proteins causing the formation of the Aβ42 domain. There are also studies suggesting that selenium supplementation may reduce the formation of amyloidosis. Research presented by Haratake et al. [141] showed that the use of a diet low in selenium in transgenic mice (Tg2576) resulted in a more than two-fold increase in the production of amyloid proteins compared to a selenium-rich diet. The use of an organic form of selenium (Sel-Plex yeast) can reduce the formation of Aβ and minimise the oxidation process of nucleic acids. Moreover, the presented research results confirm the role of selenium as a potential therapeutic agent in neurological disorders [142]. Song et al. [143] demonstrated that selenium-enriched yeast (*Saccharomyces cerevisiae*) reduces β-amyloid formation and regulates autophagy in a mouse model (3 × Tg AD) of Alzheimer’s disease. It should be noted that lack of selenium intake or its negligible amount may cause a number of unpleasant ailments. Selenium is a compound of interest in the treatment and prevention of AD and amyloids due to its role in antioxidant defence [110, 144]. According to the data presented by Rayman [136], yeast enriched with selenium is able to increase the activity of selenoenzymes [145, 146]. The expression of selenoproteins, an example of which is glutathione peroxidase, selenoprotein P, reaches its highest value with a moderate intake of this element [147, 148]. Additionally, Zhang et al. [149] showed that yeast enriched with selenium alleviates the condition of people suffering from cognitive impairment. Additionally, the selenium yeast *S. cerevisiae* reverses synaptic deficits and ameliorates tau pathology by inhibiting glycogen synthase kinase-3β (GSK-3β) activity in a triple transgenic mouse model of Alzheimer’s disease. Therefore, when discussing the role of selenium, it should be mentioned that it is a factor supporting the immune response and increases the concentration of glutathione and antioxidant enzymes [150–152], which, in turn, may support the treatment of neurodegenerative diseases [153, 154].

Moreover, the use of *S. cerevisiae* in research on ND is attributable, in part, to its possession of many endogenous proteins within its cellular structure, i.e. Sup35 protein and Swi1 protein, which form amyloid. In *S. cerevisiae*, amyloids are formed by endogenous proteins associated with transgenerational inherited epigenetic traits. Their transfer includes, among others, prions, i.e. infectious protein particles. This leads to the loss of proper functions of endogenous proteins, resulting in the formation of amyloids. ND research using *S. cerevisiae* has facilitated and accelerated the process of discovering potential therapeutic agents [132, 155].

Although yeast is a widely used model in understanding many disease mechanisms, it also has its shortcomings. *S. cerevisiae* is a single-celled organism, which in turn complicates research on the analysis of aspects of neurodegenerative diseases that focus on multicellularity and intercellular interactions, e.g. immune and inflammatory response, axonal transport, and synaptic transmission [156]. *S. cerevisiae* is a suitable model to study some of the mutations that arise during the Hailey-Hailey genetic disease, the aetiology of which is poorly understood. Hailey-Hailey disease is an autosomal dominant disorder in which there is a loss of cohesion between keratinocytes in the basal layers of the skin caused by ATP2C1 mutations. hSPCA1 protein, encoded by the *ATP2C1* gene, is a Ca^2+^/Mn^2+^ ion pump located in the Golgi apparatus. It is currently unknown why mutations in *ATP2C1* mainly affect the skin, causing the clinical phenotype. The yeast orthologue, *PMR1*, encodes a functional homologue of the ATP2C1 gene. Therefore, the use of *S. cerevisiae* genes may be useful in studying the functionality of mutant *ATP2C1* alleles occurring in humans [157].

Yeast has also been widely used in the study of various metabolic disorders, i.e. mechanisms of lipotoxicity linked with non-esterified fatty acids (NEFA), which may lead to the development of type 2 diabetes. Beta cells of the pancreas lose their function due to impaired insulin secretion, which in turn is the main cause of the development of type 2 diabetes. It is believed that a high level of NEFA in the plasma is a characteristic feature of the development of this pathology [158]. This entire process is referred to as lipotoxicity. Activation of the lipotoxicity mechanism begins when the metabolism of fatty acids in the cell is disturbed. This happens when stress is activated in the endoplasmic reticulum (ER). Saturated fatty acids (SFAs) are responsible for this process. SFAs show high toxicity in response to stress. Stress can be countered by the unfolded protein response (UPR), the main purpose of which is to help the ER cope with the accumulation of misfolded proteins [159, 160]. The destruction of the UPR mechanism of action may be a consequence of long-term exposure to SFAs, which in turn leads to the initiation of cell death. Literature data indicate that the yeast *S. cerevisiae* undergoes ER stress under lipotoxic conditions. Hence, yeast genetics may contribute to understanding the mechanisms in which the influence of saturated fatty acids on ER function plays a major role [161].

Furthermore, the development of type 2 diabetes in humans is associated with the formation of cytotoxic amyloid fibrils, which consist of islet amyloid polypeptide (IAPP), also called amylin. This polypeptide is produced by the beta cells of the islets of Langerhans, where it is then released into the bloodstream along with insulin. In the study conducted by Rencus-Lazar et al. [162], the yeast *S. cerevisiae* was used as a model organism to identify the key molecular mechanisms leading to the development of type 2 diabetes. The yeast model overexpressed six genetically encoded IAPP monomers, which then showed activation of the ER stress response and unfolded protein. Overexpression and deletion screening helped identify a peptidase, i.e. *Ste24*, which is a potent suppressor of IAPP toxicity. The homologue of *Ste24* is the peptidase *ZMPSTE24*, which occurs in humans. These peptidases play a key role in removing trapped polypeptides from the ER translocon. Therefore, overexpression of *Ste24* led to a reduction in IAPP-induced ER stress [162].

Research on *S. cerevisiae* has helped expand knowledge about important features of cancer development, i.e. aneuploidy and chromosome instability. Yeast has been shown to be an important model organism for identifying and studying new compounds, mechanisms, and applications in anticancer research. However, yeast models are still of limited use in the study of cancer development. These limitations are related to insufficient cell wall permeability and the lack of crucial human proteins that play an important role in anticancer research, e.g. tumour suppression, drug metabolism, and apoptosis. There is also a lack of research on some aspects of cancer, i.e. tissue invasion, angiogenesis, and cancer metastasis [129].

One of the most common cancers in the world is colorectal cancer, the most common form of which is hereditary non-polyposis colorectal cancer (Lynch syndrome). Lynch syndrome involves mutations in genes that encode proteins that are involved in DNA mismatch repair. Mutations in the human *hMSH2* and *hMLH1* genes are responsible for most cases of hereditary non-polyposis colorectal cancer. Mechanistic studies of DNA mismatch repair have revealed similar processes and functional protein complexes in yeast and human cells. The homologue of the human *hMSH2* and *hMLH1* genes is the yeast *MSH2* and *MLH1* genes, respectively [163]. Therefore, the isolation of genes with the same phenotype in yeast in which mutations occur will help to better understand some aspects of the development of colorectal cancer [164].

One of the rarest groups of inherited diseases are congenital disorders of metabolism, resulting from mutations in genes encoding various metabolic enzymes. During these disorders, the body is unable to convert the food consumed into energy properly, which in turn leads to the accumulation of the appropriate metabolite substrate. Literature data report that adenine has properties similar to amyloid in vitro, creating archetypal nanofibres that may cause the development of metabolic disorders. To test the accumulation of adenine in vivo, the yeast *S. cerevisiae* was used as a model organism. In humans, genes involved in the adenine rescue pathway can contribute to the development of metabolic disorders, such as mutations in adenosine deaminase (ADA) and adenine phosphoribosyl transferase (APRT). These mutations, in turn, can prevent the accumulation of adenine and its derivatives, leading to disturbances in the functioning of various organs. In order to mimic the accumulation of metabolites in yeast cells, the orthologs of the human *ADA* and *APRT* genes, i.e. *APT1* and *AHH1*, were disrupted [162].

In studies conducted by Porat et al. [165], it was shown that the use of tannic acid in yeast containing high levels of adenine did not reduce its level. This may indicate that disorders in the functioning of superorgans may result not only from the presence of high levels of adenine but also from the presence of toxic structures, e.g. amyloid fibres [165]. Various studies using key regulators that play an important role in maintaining metabolic homeostasis in the cell have made yeast research essential for understanding the basic principles of cell biology, and they are also of great importance for understanding diseases and protecting human health [125].

*S. cerevisiae* has made a significant contribution to the study of the mechanisms of ageing in humans. Ageing is the most important risk factor for age-related diseases such as Alzheimer’s, Parkinson’s, and Huntington’s [166]. Reducing the availability of glucose or amino acids (asparagine and glutamate) during yeast cultivation extends the replicative and chronological lifespan [167–169]. There is a high similarity between the genes of this microorganism and human genes, where of the 414 essential yeast genes, approximately 47% are orthologs of human genes. For example, *Sirtuin 2* in *S. cerevisiae* is an ortholog of the human *SiRT1* gene, which is an important sensor of the metabolic state of cells [125]. Sir2 is a nicotinamide adenine dinucleotide (NAD^+^)-dependent histone deacetylase that plays a key role in gene silencing and extends the lifespan of *S. cerevisiae* [170]. Overexpression of *Sir2* leads to inhibition of the accumulation of circular DNA molecules during homologous recombination in ribosomal DNA genes, which, in turn, positively affects lifespan. Lifespan extension in *S. cerevisiae* may be achieved by caloric restriction, during which NAD^+^ levels are higher, which, in turn, ultimately leads to greater *Sir2* activity. With the molecular properties of *Sir2* now discovered, more information about polyphenolic resveratrol can be provided [140]. Resveratrol has antioxidant and anti-inflammatory properties, thanks to which it can inhibit biochemical changes associated with the initiation, promotion, and progression of cancer [171]. This compound is contained in red wine and grapes and can prolong life thanks to its ability to activate *Sir2* in *S. cerevisiae* [172, 173]. It cannot be ruled out that some aspects of ageing may be specific to yeast. However, there is a good chance that genes regulating longevity in yeast will provide more knowledge about the ageing process of human cells, which will lead to the prevention of the development of various diseases [167]. Recently, in a study by Cereghetti et al. [174], it was shown that under stress conditions, yeast produces functional amyloids with a reversible mechanism using the pyruvate kinase Cdc19. These integrative results may indicate the possibility of protein aggregation as a physiological rather than pathological mechanism. To confirm this hypothesis, further studies should demonstrate whether these physiological and pathological aggregates share common mechanisms of amyloid formation and removal. This, in turn, could help treat amyloidosis-related diseases [162, 174].

Currently, there is no treatment that can prevent the development of diseases associated with amyloidosis, i.e. neurodegenerative diseases. However, a major discovery in recent years is that bacteriophages (bacterial viruses) can reverse the aggregation of misfolded proteins in the brain that occur during Alzheimer’s disease. In a study by Frenkel et al. [175], phage display was used to identify the human β-amyloid epitope that produces antibodies against amyloid plaques. The results of these studies were unexpected because the phage reversed the aggregation of misfolded proteins [176].

In another study conducted by Dimant et al. [177], similar results were obtained, where the bacteriophage caused the disaggregation of alpha-synuclein, which is a neuronal cytoplasmic protein. This protein is referred to as a non-amyloid component of amyloid plaques and occurs in presynaptic terminals in the central nervous system [177, 178]. Alpha-synuclein, similar to β-amyloid, can aggregate into amyloid fibres, which disrupt neuronal function in the brain. During the incubation of the bacteriophage with a solution containing amyloidogenic fibres, their degradation was observed. Hence, the use of bacteriophages may provide a new approach to the treatment of amyloidosis-related diseases [176].

In conclusion, it should be noted that yeast cells are a promising microorganism used in many studies aimed at understanding the functioning of basic cellular processes. Further research should aim to clarify the activity of the early mechanism responsible for protein misfolding and assembly and the toxicity of amyloidosis. The issues related to understanding and identifying new amyloids emerging in cells are also very important. The eukaryotic microorganisms used in the research fulfil the expectations placed on them to develop an effective strategy in the treatment of various disease states, which will consequently contribute to the development of science.

## Food Protein Amyloidosis

In recent years, research on food proteins in food nanotechnology has gained wide interest [179]. Amyloid fibres can be divided into pathological (associated with various human diseases) and non-pathological, such as food proteins. These proteins have been found to be useful in the food industry. Every day, humans consume large amounts of protein nanostructures, which are quickly broken down in the gastrointestinal tract [180]. Due to their amazing physicochemical properties, protein-based food is used in many industrial branches, including medicine, environmental sciences, and nanotechnology [181]. The use of dietary proteins in food may enhance gelling, foaming, and emulsifying properties due to amyloid fibres having better functionality than other protein aggregates. Due to the possibility of the formation of amyloid protein structures in food proteins, these structures may contribute to reducing the level of animal protein while maintaining the quality of the product [182]. Amyloid fibres are formed through the transformation of soluble proteins and peptides into protofibrils and mature fibres. This process, which results in highly ordered structures, occurs under the influence of environmental factors, i.e. heat and radiation [183].

The best-known dietary proteins that form amyloid fibres under various conditions are β-lactoglobulin. The resulting fibres can be used as additives that change the texture of food, as well as its digestibility and gelling properties. Amyloid aggregates from bovine β-lactoglobulin or obtained from whey protein isolate are effective gelators and good iron carriers [184]. Perhaps in the future, they can also serve as effective thickeners, where the rheological properties of the solution can be changed by the shape factor of the amyloid fibres without changing the mass or caloric content of the food [180]. The source of amyloid fibres may be egg white, bovine serum albumin, whey protein, or soy protein, which form at low pH and low ionic strength [183]. It is worth noting that amyloid-like aggregates occurring in plant seeds are involved in the stabilisation of storage proteins [184]. Zhang and Dee [185] reported that legume seed storage proteins can be used to create amyloid fibrils. Such activities may improve their functionality for use in food. In addition, due to the extensive occurrence of functional groups and the high ratio of surfaces to volume, amyloid fibres are capable of carrying bioactive substances and nutrients [179, 181]. Such structures are increasingly used in encapsulation technology [186]. Another source of proteins that create amyloid fibres is filamentous fungi. These include amphiphilic hydrophobins, naturally occurring in the fungus *Neurospora crassa* [39]. Thanks to hydrophobins, amphiphilic monolayers form on the surface of fungal spores. These layers provide a hydrophobic coating to the hyphae and spores, which in turn helps prevent water penetration into the media [180]. Due to their amphiphilic properties, these proteins can be used as surfactants, for example, in creating an air-filled emulsion to replace fat in food products [187].

There are some amyloid fibres of food proteins that have toxic and pathogenic effects. An example is kappa-casein, found in cow’s milk, which forms a cytotoxic amyloidogenic protein. However, the presence of beta-casein may inhibit the cytotoxicity of kappa-casein. Therefore, the use of a homogeneous preparation containing kappa-casein amyloid fibres may be dangerous for humans [180]. Rising et al. [188] showed that meat from Swedish and Italian cattle intended for human consumption often contains AA amyloid. The authors showed that the consumption of such meat may be a risk factor for diseases in humans. Bovine AA fibrils cross effectively with human β-amyloid peptide, which is associated with Alzheimer’s disease. Similar research results were shown by Vaneyck et al. [189]. The authors demonstrated the potential for cross-spreading of aggregation of amyloid fibrils formed from proteins found in food to alpha-synuclein. Based on the presented literature analysis, a comparable course of changes occurring between alpha-synuclein and amyloid proteins derived from food products can be concluded. The consequence of such processes is the induction of aggregation of proteins found in the brain associated with Parkinson’s disease. Currently, only a few studies have been conducted to understand the direct toxicity of non-disease-related amyloid fibrils in cell cultures [190–192]. Therefore, the presented scientific topic requires further research. In order to use amyloid fibres in the food industry, it is necessary to understand the physiological processes, i.e. the process of fibre digestion while passing through the stomach and intestines. Literature data indicate that beta-lactoglobulin from cow’s milk forms amyloid fibres that are digested by pepsin in vitro. Moreover, the resulting hydrolysates are capable of creating new fibres with different morphologies. According to research conducted by Watt et al. [193], it was found that one of the characteristic features of amyloidogenic fibres is their high resistance to hydrolysis by proteases, i.e. trypsin and proteinase K. Hence, there is a need for further studies of the in vitro digestibility of amyloidogenic fibres [191]. In a study conducted by Cui et al. [194], it was shown that AA amyloidosis in mice is accelerated by the administration of extracted amyloid fibres. AA amyloid deposits are most often found in birds exposed to environmental stress and force-fed during the production of foie gras (Strasbourg pâté) [195]. Moreover, oral consumption of foie gras extract containing AA amyloidosis has an amyloid-enhancing factor in transgenic animals. It is worth noting that in the food industry, the properties of amyloid fibres have been used to obtain microcapsules. Such structures can be used to deliver bioactive substances and nutrients to various products [179]. Therefore, concerns arise regarding human consumption of foods containing amyloid proteins, which require further research [194].

A diet rich in selenium can help maintain good health. According to information presented by Cardoso et al. [196], people suffering from AD have much lower concentrations of selenium in plasma or red blood cells compared to healthy people. Fu et al. [197] showed that the use of fish oil with the addition of selenium and zinc inhibits the activity of β- and γ-secretase in the β-amyloid precursor protein enzymatic pathway. The consequence of these processes is a reduction in the production of Aβ1–40. Additionally, the latest literature data [198, 199] indicate that excessive selenium supplementation may also increase the risk of various disease states (type II diabetes). Appropriate knowledge of the rules for dosing this element is crucial in the context of health and safety. Chen et al. [200] concluded that selenium-enriched foods can inhibit the occurrence of inflammation and oxidative stress. The presented data provide evidence supporting the potential use of selenium materials as a new strategy in the fight against AD. Therefore, it is very important to conduct further high-quality scientific research aimed at determining the impact of selenium, its chemical forms, and its dose on the processes of amyloid formation.

## Conclusion

It should be emphasised that further research is necessary to understand the nature of selenium and its impact on the metabolic processes taking place in cells. Such assumptions are very important in solving the presented problems of oxidative stress and the formation of β-amyloid fractions in cells. Moreover, determining the functioning of the entire set of mechanisms and the impact of selenium on the processes occurring in living cells is very important from the point of view of the possibility of inhibiting the development of various pathological conditions, an example of which is Alzheimer’s disease (AD). The role of selenium in amyloid formation remains ambiguous. Therefore, understanding the significance of this element and its impact on the entire process of the formation of these protein structures at the molecular level, especially using yeast cells as a model organism in biotechnological research, is very important. Moreover, expanding knowledge about amyloidosis and the functioning and use of food proteins will enable their deeper interpretation in the future and increase the possibilities of designing new food products. As research in this area progresses, collaborative efforts between scientific disciplines will enable the development of innovative therapeutic interventions that could revolutionise the treatment of diseases caused by the formation of amyloid fibres.

## Data Availability

No datasets were generated or analysed during the current study.
